# Measuring the Impact of Targeting FcRn-Mediated IgG Recycling on Donor-Specific Alloantibodies in a Sensitized NHP Model

**DOI:** 10.3389/fimmu.2021.660900

**Published:** 2021-06-02

**Authors:** Miriam Manook, Walter J. Flores, Robin Schmitz, Zachary Fitch, Janghoon Yoon, Yeeun Bae, Brian Shaw, Allan Kirk, Melissa Harnois, Sallie Permar, Alton B. Farris, Diogo M. Magnani, Jean Kwun, Stuart Knechtle

**Affiliations:** ^1^ Duke Transplant Center, Department of Surgery, Duke University Medical Center, Durham, NC, United States; ^2^ Massbiologics of the University of Massachusetts Medical School, Boston, MA, United States; ^3^ Human Vaccine Institute, Duke University Medical Center, Durham, NC, United States; ^4^ Department of Pathology, Emory School of Medicine, Atlanta, GA, United States

**Keywords:** FcRn, IgG, alloantibody, sensitization, non-human primate (NHP)

## Abstract

**Background:**

In transplantation, plasmapheresis and IVIg provide the mainstay of treatment directed at reducing or removing circulating donor-specific antibody (DSA), yet both have limitations. We sought to test the efficacy of targeting the IgG recycling mechanism of the neonatal Fc receptor (FcRn) using anti-FcRn mAb therapy in a sensitized non-human primate (NHP) model, as a pharmacological means of lowering DSA.

**Methods:**

Six (6) rhesus macaque monkeys, previously sensitized by skin transplantation, received a single dose of 30mg/kg anti-RhFcRn IV, and effects on total IgG, as well as DSA IgG, were measured, in addition to IgM and protective immunity. Subsequently, 60mg/kg IV was given in the setting of kidney transplantation from skin graft donors. Kidney transplant recipients received RhATG, and tacrolimus, MMF, and steroid for maintenance immunosuppression.

**Results:**

Circulating total IgG was reduced from a baseline 100% on D0 to 32.0% (mean, SD ± 10.6) on d4 post infusion (p<0.05), while using a DSA assay. T-cell flow cross match (TFXM) was reduced to 40.6±12.5% of baseline, and B-cell FXCM to 52.2±19.3%. Circulating total IgM and DSA IgM were unaffected by treatment. Pathogen-specific antibodies (anti-gB and anti-tetanus toxin IgG) were significantly reduced for 14d post infusion. Post-transplant, circulating IgG responded to anti-FcRn mAb treatment, but DSA increased rapidly.

**Conclusion:**

Targeting the FcRn-mediated recycling of IgG is an effective means of lowering circulating donor-specific IgG in the sensitized recipient, although in the setting of organ transplantation mechanisms of rapid antibody rise post-transplant remains unaffected.

## Introduction

In transplantation, plasmapheresis (plasma exchange, PEX, or double filtration plasmapheresis, DFPP) has been one of the mainstays of many protocols aiming to remove circulating donor-specific antibody (DSA). For sensitized patients, indications for therapy are prophylactic removal of DSA perioperatively to facilitate transplantation (1, 2), or as treatment for antibody-mediated rejection (AMR) (3). Despite wide utility, this treatment is limited by efficacy (4), and although major complications are rare, the therapy is non-specific and risks off-target effects, such as removal of circulating soluble coagulation factors thus increasing bleeding risk (5), as well as concurrent pharmacological therapies (6), necessitating additional treatment.

In the clinical setting of HLA-incompatible transplantation, the introduction of IdeS (imlifidase) has permitted highly sensitized patients to receive a deceased donor kidney against whom they have a positive initial crossmatch by cleaving soluble IgG at the lower hinge region, thus generating F(ab) and Fc fragments and swiftly reducing levels of circulating DSA shortly before transplantation to a negative crossmatch, in the absence of the deleterious effects of plasmapheresis (7, 8). In addition, further evidence suggests that IdeS is also effective in cleaving IgG when cell bound to the IgG subtype BCR (B cell receptor) complex, resulting in an inhibitory effect on antibody-secreting cells (ASCs) (9). Despite this success, the utility of this treatment may be limited by the emergence of anti-drug antibodies (ADA), which would reduce efficacy and therefore prevent repeated use of IdeS (10).

An alternative biologic mechanism to reduce levels of circulating antibody is to target the neonatal Fc receptor (FcRn). This receptor is expressed in most tissues, but is reliably found in endothelial cells, and antigen presenting cells (APCs) such as macrophages/monocytes, dendritic cells, and B cells (11, 12). Related to the MHC structure, but incapable of itself presenting IgG to T cells, the neonatal FcRn is so called as it is necessary for the passive transfer of maternal antibody to the neonate. Despite the infant name, this receptor persists throughout life and remains an important mechanism by which circulating IgG levels are maintained. IgG captured on the cell surface by the FcRn is taken up by intracellular vesicles under mildly acidic conditions to be subsequently re-released into serum (13, 14). A high affinity monoclonal antibody that targets the FcRn, Rozanolixizumab, has been developed and tested in cynomolgus monkeys, as well as humans (15, 16). Therapeutically, blocking the FcRn is actively being investigated for its role in the treatment of IgG-mediated diseases, such as myasthenia gravis, and primary immune thrombocytopenia (17), and has been reported as a potential agent in the setting of AMR in transplantation both for its direct effect on lowering circulating antibody levels, and as a mechanism to be exploited with respect to maintaining circulating drug levels of IgG-based therapeutics (18, 19). However, it has not been tested in rhesus macaque models of sensitization or IgG-mediated pathology. In the present study, we hypothesized that rhesus specific anti-FcRn mAb could provide a safe and effective pharmacological method to reduce circulating DSA, as well as total IgG levels, and thereby facilitate kidney transplantation in allosensitized recipients.

## Materials and Methods

### Animals, Surgical Procedures, and Drug Treatments

All animal care and procedures were conducted in accordance with National Institutes of Health (NIH) guidelines and were approved by the Duke University Institutional Animal Care and Use Committee (Duke IACUC# A153-18-06). Six juvenile (3–5 year old) male NHPs (*Macaca mulatta*) were obtained from Alphagenesis (Yemassee, SC). Donor and recipient pairs are created between animals with maximal MHC class-1 and -2 mismatching. Donor-recipient NHP pairs were sensitized to each other with two sequential full-thickness skin transplants performed as previously described (20). A month after skin transplantation, when the anti-donor response is stabilizing following a peak. six animals were treated with a single ‘test-dose’ of 30mg/kg of anti-RhFcRn mAb (anti-FcRn [RozR1LALA], a LALA-mutated rhesus IgG1 chimeric of Rozanolixizumab, NIH Nonhuman Primate Reagent Resource Cat# PR-0001, RRID: AB_2888630). Peripheral blood sampling was performed to monitor full recapitulation of circulating antibody response at 0, 1, 4, 7, 13, 20 and 27 days post-test dose. The administration of non-native therapeutic monoclonal antibodies (mAbs) can induce anti-drug antibody (ADA) responses in humans (21) and nonhuman primates (NHP) (22) which can affect drug pharmacokinetics and efficacy, limiting the informative value of NHP studies. Therefore, to mitigate the impact of ADA in NHP experiments, the nonhuman primate reagent resource (nhperagents.org) engineered a ‘primatized’ mAb against FcRn (anti-FcRn [RozR1LALA], a LALA-mutated rhesus IgG1 chimeric of Rozanolixizumab).

A donor-recipient pair of animals (n = 2) received swopping kidney transplantation from their maximally mismatched skin donor, with bilateral native nephrectomies at six weeks after test dosing as previously described (23). For kidney transplantation, anti-RhFcRn mAb was administered at 60mg/kg IV on day –5, 0, and +5 day of kidney transplantation. All transplanted NHPs received induction therapy with 20 mg/kg IV rhesus ATG (Anti-rhesus thymocyte [rhATG7] - Lot 7, rhATG#7, NIH NHP Reagent Resource Cat # PR-1077, RRID: AB_2819339) in 5 divided doses (POD 0 to 4, 4mg/kg daily). Maintenance immunosuppression after kidney transplant consisted of intramuscular (IM) tacrolimus (Astellas Pharma, Northbrook, IL) twice daily with the dose adjusted to maintain trough levels at 8–12 ng/ml, 30 mg/kg by mouth of mycophenolate mofetil (MMF) for oral suspension (Genentech, San Francisco, CA) twice daily, and methylprednisolone (Pfizer, New York, NY) tapered from 15 mg/kg on day of transplant and then a halved dose daily until a maintenance dose of 0.5 mg/kg was reached and continued until the end point. A subcutaneous dose of 6 mg/kg of ganciclovir (Fresenius Kabi, Lake Zurich, IL) was administered daily as rhesus cytomegalovirus (rhCMV) reactivation prophylaxis.

Comparison in survival and anticipated DSA levels were made between these anti-FcRn mAb treated animals, and previously established historic controls who received the same induction and maintenance immunosuppression, but no desensitization therapy prior to kidney transplantation (24).

In addition, evidence of circulating RhATG levels in Rh anti-FcRn mAb receiving a kidney transplant were compared with time matched (d5 after first ATG dose) animals who received either 20 mg/kg IV rhesus ATG (RhATG#5, n = 4 + RhATG#6, n = 4, NIH NHP Reagent Resource) in 5 divided doses without kidney transplantation (POD 0 to 4, 4mg/kg daily, or rabbit ATG as given to humans (rATG, n= 4), (25).

### Anti-FcRn Monoclonal Antibody Quantitation by ELISA

The presence of the anti-FcRn in sera was quantitated *via* ELISA. Purified recombinant rhesus FcRn soluble protein was used to coat plates overnight at 1 µg/µL in PBS (pH 8.0, 4°C). ELISA plates were then washed three times with PBST and blocked with 300uL of non-fat dry milk in PBS (pH 8.0) for 1 h at 37°C. The standard curves were generated with the recombinant anti-FcRn antibody starting at a concentration of 10 µg/µL. Sample dilutions were done in PBS (pH8.0), 5% non-fat dry milk, and 10% plasma with a total volume of 100 uL to each well. Serum samples with unknown amounts of anti-FcRn were serially diluted 2-fold with an initial dilution of 1:10, then added to wells and incubated for 1 h at 37°C. ELISA plates were washed three times with PBST and 100 µL of HRP conjugated anti-human kappa (Southern Biotech) was added to each well and incubated for 1 h at 37°C. The ELISA plate was then washed three times with PBST and developed by adding 100 uL of TMB substrate at room temperature for 2–3 min, stopped and read at 450 nm.

### Quantitative Measurement of Anti-Drug Antibody (ADA) Response in Serum

Anti-drug antibody (ADA) responses were evaluated by ELISA against the recombinant anti-FcRn antibody (1 µg/µL coated overnight at 4°C in PBS pH 7.4). ELISA plates were then washed three times with PBST and blocked with 300uL of non-fat dry milk in PBS (pH 7.4) for 1 h at 37°C. Sample dilutions were done in PBS (pH 7.4), 5% non-fat dry milk with a total volume of 100 uL to each well. Serum samples were serially diluted 2-fold with an initial dilution of 1:10, then added to wells and incubated for 1 h at 37°C. ELISA plates were washed three times with PBST and 100 µL HRP conjugated anti-lambda (Southern Biotech) was added to each well and incubated for 1 h at 37°C. ELISA was then washed three times with PBST, developed with TMB, and read at 450 nm. Samples were considered ADA-positive if the absorbance of the sample was 2 times higher than the pretreatment samples.

### Measurement of DSA

As described previously, DSA levels were determined by flow cytometric crossmatching using donor splenocytes or PBMCs, incubated with recipient serum from serial blood draws (26). DSA titer measurement was made on serum samples which were stored at –80°C and batched for analysis. For IgG measurement, samples were serially diluted (1:50) in each run, while for IgM measurements, neat sera were used. No serum, ‘naïve’ (pre-sensitization) as well as ‘peak’ (2 weeks following second skin transplantation) sensitization samples were run as negative and positive controls. Donor PBMCs or splenocytes were incubated with recipient serum, washed, and then stained with FITC-labeled anti-monkey IgG (Sera Care, 5210-0216), or FITC-labelled anti-monkey IgM (Sera Care, 5230-0423), anti-CD20 mAb (2H7), anti-CD3 mAb (SP34–2) (both BD Bioscience), and Live/Dead Fixable Blue staining (Life Technologies, Carlsbad, CA). The mean fluorescence intensity (MFI) of anti-monkey IgG or IgM on T or B cells was measured on BD LSRFortessa (BD Biosciences) and analyzed using FlowJo software version 9 or 10 (Tree Star). Results were expressed as either MFI, or MFI fold-change from the pre-sensitized (naïve) time point. A MFI value lower than the neat naive sample was considered negative.

### Total IgG Measurements

Measurement of IgG in sera was done by surface plasmon resonance (SPR) using a protein G chip and calibration-free concentration analysis (CFCA) (27). Concisely, the serum samples were initially diluted 1:10000 in HEPES-Buffered Saline (HBS) and then serially diluted 2-fold in HBS and loaded into a the Biacore T200 SPR equipment. CFCA calculations were done using software using an average molecular weight of 150 kDa.

### Total IgM Measurements

Measurement of IgM in sera was measured by Human IgM ELISA Kit (Bethyl Laboratories, Inc. E88-100). The kit contains an ELISA plate pre-coated with anti-human IgM antibodies. The 8 standard solutions were prepared by reconstituting a vial of 750 ng of human IgM in 1.0 ml of 1X Dilution Buffer B and serially diluting by 3-fold. The sera samples were prepared by diluting 10 µL of sera in 990 µL of 1X Dilution Buffer B, and repeating the dilution again to yield a total of 1:10000 dilution. Wells in the ELISA plate were loaded with 100 µL of standard solutions or diluted sera samples. The plate was incubated at room temperature (20–25°C) for 1 h. ELISA plate was then washed four times with 1X Wash Buffer (300µL/well) and 100 µL of biotinylated detection antibody was added to each well, to be incubated at room temperature for 1 h. The ELISA plate was then washed four times with 1X Wash Buffer and the plate was incubated for 30 min at room temperature after adding 100 µL of streptavidin-conjugated horseradish peroxidase (SA-HRP) in the wells. Plates were then washed four times with 1X Wash Buffer (300µL/well) and the plate was developed for 30 min at room temperature in the dark after adding 100 µL of 3,3′,5,5′-Tetramethylbenzidine (TMB) in each well. After the reaction was stopped with 100 µL of Stop Solution, the plate was read using a plate reader at 450 nm.

### ATG Quantitation by ELISA

The presence of ATG antibodies in sera was measured by ELISA using rabbit-specific antibodies. ELISA plates were coated overnight at 4°C with 100 µL at 1 µg/µL of Goat anti-Rabbit IgG Antibody (EMD Millipore) in PBS (pH 7.4). ELISA plates were then washed three times with PBST and blocked with 300 uL of non-fat dry milk in PBS for 1 h at 37°C. A standard curve was generated with rhesus ATG starting at a concentration of 10 µg/µL and doing a 2-fold serial dilution. Successively, on the same plate, serial dilutions of 2-fold was done on rhesus serum in PBS with 5% non-fat dry milk. ELISA plates were washed three times with PBST and 100 µL of HRP-conjugated goat anti-rabbit IgG (H+L) (Jackson Immuno Research) was incubated on each well for 1 h at 37°C. ELISA plates were then washed three times with PBST and developed by adding 100 uL of TMB substrate at room temperature for 2–3 min. After the reaction was stopped, the plates were read using a spectrophotometer at 450 nm.

### Measuring Tetanus Toxoid-Specific and Rhesus gB-Specific IgG

The magnitude of tetanus toxoid-specific and rhesus gB-specific IgG responses were measured by ELISA. First, 384-well plates were coated with either 2 μg/ml of Clostridium Tetanus Toxoid (Creative Diagnostics) or 1.5 μg/ml of RhCMV gB, diluted in 0.1 M sodium bicarbonate buffer, pH 9.6. Coated plates were incubated at 4°C overnight. Plates were then washed one time with wash buffer (95% DI water, 4% 25X PBS, 1% Tween-20), blocked with 40 µl blocking buffer per well (4% whey, 15% goat serum, 0.5% tween 20, 80.5% 1X PBS), and incubated at 4°C overnight. Samples were diluted in blocking buffer at 1:5 for tetanus toxoid ELISA and 1:30 for Rhesus gB ELISA and serially diluted 1:3 in a 96-well plate (CSL Behring). A known seropositive sample was used as a positive control for the gB ELISA and the WHO tetanus standard was used as a positive control for the tetanus toxoid ELISA. After blocking, plates were washed one time and 10 µl of diluted sample were added to the plate in duplicate. Plates were incubated for 2 hours at RT. Secondary antibodies (Mouse anti-Monkey IgG-HRP, Southern Biotech; Goat anti-Human IgG-HRP, Jackson ImmunoResearch) were prepared at a 1:5000 dilution in blocking buffer. After the incubation, plates were washed two times and 10 µl of diluted secondary antibody were added to each well. Plates were sealed and incubated for 1 hour at RT. Plates were then washed four times and 20 µl of room temperature SureBlue Reserve TMB Substrate (VWR) was added to each well. Plates were incubated for 10 min while shielded from light. After incubation, 20 µl of TMB Stop Solution (VWR) was added to each well. Plates were immediately read at 450 nm on the SpectroMax using the SoftMax software interface. Data are reported as area under the curve (AUC) because full sigmoidal curves were not achieved by all samples.

### Histology, Immunohistologic Analysis, and Pathologic Grading

At euthanasia, graft specimens were harvested and fixed in 10% buffered formalin before being paraffin embedded for sectioning, and staining with hematoxylin & eosin (H&E), Schiff (Periodic Acid–Schiff) or polyclonal anti-human C4d (American Research Products, Waltham, MA) prior to histologic grading. Histology specimens were evaluated in a blinded fashion by a transplant pathologist (A.B.F.) and scored based on the current Banff criteria of renal allograft pathology (28–31).

### Statistical Analyses

Statistical analyses were performed using Prism 8.0 (GraphPad Software, San Diego, CA). Data are expressed as mean±SD (error bar), and p values of less than 0.05 were considered to be statistically significant. Normally distributed data within the same treatment group but at different time points were evaluated using a two-tailed paired t test, while statistical comparisons between different groups were performed with two-tailed unpaired t test for normally distributed data or the Mann–Whitney U test for categorical data.

## Results

### Anti-Rhesus FcRn mAb and Anti-Drug Response in Sensitized NHPs

**Figure 1:**
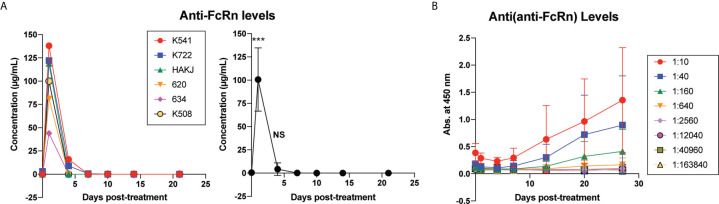
Kinetics of anti-FcRn mAb and anti-drug antibody (ADA) levels following a single dose of rhesus anti-FcRn mAb (30mg/kg IV, n=6). **(A)** Anti-FcRn mAb concentration detected by ELISA in serum post-infusion over time demonstrating individual and collective values and rapid fall in circulating mAb present. **(B)** Generation of anti-drug antibodies ADAs over time detected by ELISA. ***, < 0.001, ns, not significant.

Maximally MAMU mismatched rhesus macaque pairs underwent swapping skin transplantation as a sensitization event. One month following skin transplantation, once serum DSA had declined from a peak, 6 animals were given a single dose of 30mg/kg of anti-Rh FcRn mAb IV. Infusions were well tolerated and did not result in adverse reactions. Anti-Rh FcRn mAb was detected in serum on 1d post infusion, but was rapidly undetectable thereafter (**Figure 1A**). ADA (anti-anti-FcRn antibody) was detectable following a single dose, with maximal development at 21d post infusion (**Figure 1B**). Interestingly, ADA responses varied considerably by animal with pre- and peri-transplant dosing (**Supplemental Figures 1** and **3**).

### Effect of Anti-Rhesus FcRn mAb on Total Circulating IgG and IgM

**Figure 2:**
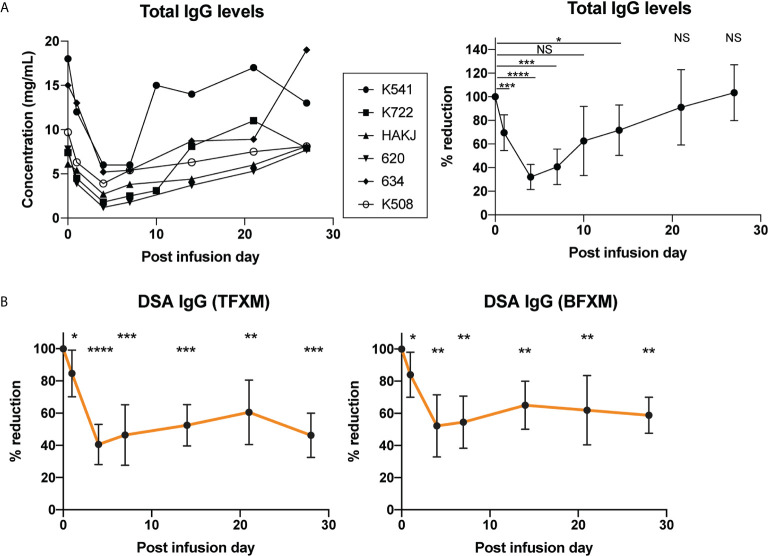
Effect of a single dose of rhesus anti-FcRn mAb (30mg/kg IV, n=6) on circulating total IgG and IgG donor-specific antibody (DSA) levels. **(A)** Total serum IgG concentration for individual animals (left). Total IgG level was reduced after anti-FcRb mAb treatment. **(B)** Percent reduction of circulating DSA IgG with TFXM and BFXM post-infusion demonstrating maximal reduction on post infusion day (POD) 4 to POD7, and persistent significant reduction to 1 month post infusion. * indicates p<0.05; ** indicates p<0.001; *** indicates p<0.005; NS indicates no statistical significance.

The total IgG was reduced for all animals, which was most maximally evident on post infusion day (PID) 4 with a mean reduction of 68±10.6% from baseline which was significant (p<0.05), although the likely nadir may have been on PID 5 (**Figure 2A**). By PID 28, the total IgG level had recapitulated back to baseline (103± 23.7% of baseline, **Figure 2A**). In contrast, while both CD3 (T cell FXCM) and CD20 (B cell FXCM) reduced significantly on PID 4 (TFXM 40.6% of baseline SD ± 12.5, p = <0.05 BFXM 52.8% of baseline SD ± 19.3, p = <0.05), the reduction was maintained at PID 28 (TFXM IgG 46.2% of baseline SD ± 13.8, p = <0.05, BFXM IgG 58.8% of baseline SD ± 11.2, p = <0.05, **Figure 2B**), suggesting a more sustained effect on DSA in contrast to circulating total IgG levels. Typically, in this model, a gradual decay of circulating DSA is observed over time. The sustained reduction of DSA observed in anti-Rh FcRn treated animals was, however, greater, compared to historical untreated controls at the same experimental time interval (see **Supplemental Figure 1**). Interestingly, the second skin transplant had the same effect in boosting serum DSA for anti-Rh FcRn treated animals, suggesting that there was no continuing effect reducing circulating DSA IgG, and that the humoral apparatus to generate DSA remained intact (see **Supplemental Figure 2**).

**Figure 3:**
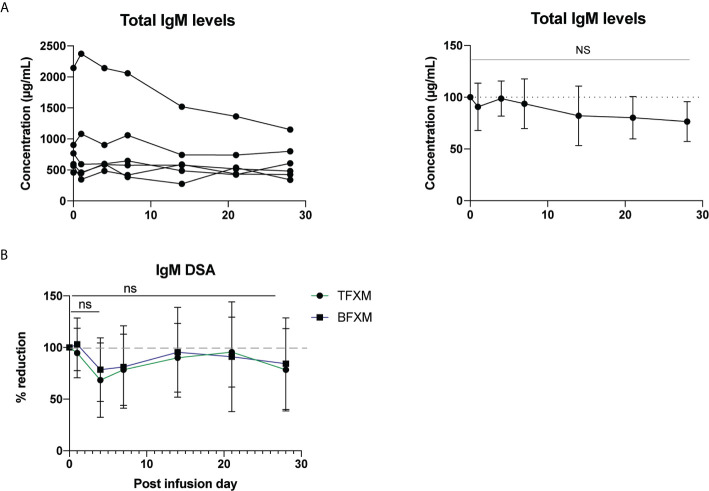
Effect of a single test dose of rhesus anti-FcRn (30mg/kg IV, n = 6) on circulating IgM. **(A)** Total serum IgM concentration by animal. Reduction of total serum IgM post infusion. **(B)** Percent reduction of circulating donor-specific antibody (DSA) IgM for CD3 (TFXM) & CD20 (BFXM) post infusion demonstrating no significant reduction for one month following infusion. NS indicates no statistical significance.

Total IgM and DSA IgM showed no evidence of significant reduction following anti-RhFcRn mAb treatment on PID 4, compared to PID 0 (TFXM IgM 68.5% of baseline SD ± 35.99, p = 0.06, BFXM 78.62% of baseline SD ± 30.8, p = 0.12, **Figure 3B**), and largely remained static over the course of the post infusion period **(** **Figures 3A, B** **).** Together, these data suggest that a single dose (30mg/kg) of anti-FcRn mAb tentatively reduces total circulating IgG and DSA IgG but is incapable of reducing the IgM isotype of DSA.

### Effect of Anti-Rhesus FcRn mAb on Pathogen-Specific IgG

**Figure 4:**
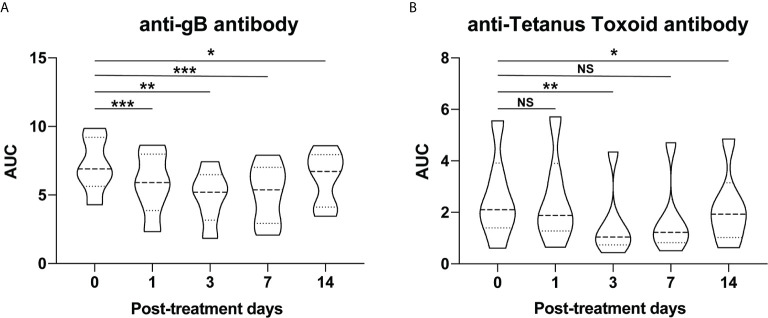
Pathogen-specific antibody changes after anti-FcRn mAb treatment. Effect of single dose of rhesus anti-FcRn mAb (30mg/kg IV, n=6) on circulating antibodies against protective immunity **(A)** CMV envelope glycoprotein B (gB) and **(B)** tetanus toxoid. * indicates p<0.05; ** indicates p<0.001; *** indicates p<0.005; NS indicates no statistical significance.

Since all rhesus macaques are vaccinated against tetanus toxoid and acquire rhesus cytomegalovirus (rhCMV) exposure in life, we measured the effect of a single dose of Rh-anti-FcRn treatment on protective immunity by measuring antibodies against CMV envelope glycoprotein B (gB) and tetanus toxoid (26). Unsurprisingly, as with the total and DSA IgG, a significant reduction in circulating pathogen-specific antibody was measured which reached nadir at PID 7 for anti-gB (rhesus CMV) antibody, and PID 4 for anti-tetanus toxoid antibody, **Figures 4A, B**. These data demonstrate the non-selective nature of anti-FcRn mAb in blocking the recycle of IgG antibodies, regardless of their specificity.

### Anti-FcRn as Antibody Removal Treatment in the Context of Sensitized Kidney Transplantation

**Figure 5:**
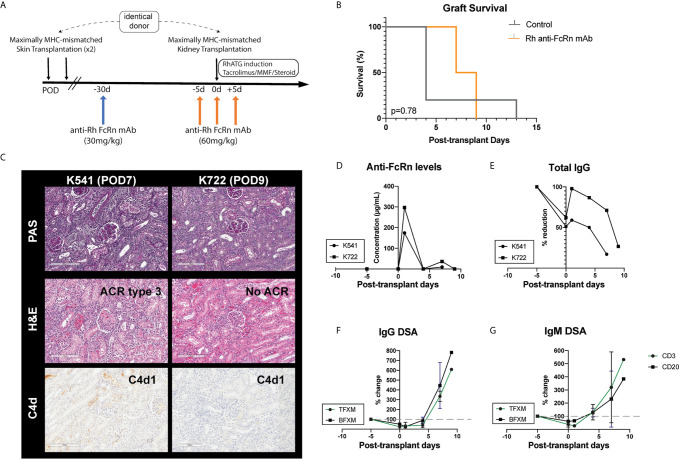
Desensitization with anti-FcRn mAb for kidney transplantation in a highly sensitized non-human primate (NHP) model. **(A)** Schematic representation of dosing strategy and immunosuppression regimen indicating single test dose (30mg/kg IV), followed by administration of 60mg/kg IV in the peri-transplant setting. **(B)** Graft survival of transplanted animals treated with anti-FcRn mAb compared to control (no desensitization). **(C)** Kidney allograft histopathology (PAS, H&E, and C4d staining) from anti-FcRn mAb-treated animals demonstrating evidence of capillaritis, glomerulitis, and C4d deposition. K541 also demonstrated evidence of acute cellular rejection. **(D)** Measurement of circulating anti-FcRn mAb detectable in serum. **(E)** Reduction of total serum IgG following administration of anti-FcRn mAb. **(F)** Percentage change pre-transplant (d–5) of circulating DSA IgG with TFXM and BFXM. **(G)** Percentage change from pre-transplant (d–5) of circulating DSA IgM with TFXM and BFXM post-infusion.

Given the efficacy of a single dose of Rh anti-FcRn, we tested a higher dose (60mg/kg, IV) in the setting of allosensitization kidney transplant in order to test whether a more profound reduction of DSA could be achieved. Two animals received Rh anti-FcRn 5 days prior to kidney transplantation, and again on the day of kidney transplantation, following initiation of the first dose of RhATG, as well as on POD5 (**Figure 5A**). Compared to previously reported control animals treated with the same induction and maintenance regimen who received no desensitization (24), there was no prolongation in survival with rh anti-FcRn mAb (**Figure 5B**). Histopathology of the kidney allograft demonstrated evidence of acute AMR in both anti-FcRn treated animals, with C4d deposition, and glomerulitis and peritubular capillaritis (**Table 1**). One animal also had evidence of acute cellular rejection, in addition to TMA-like features with glomerular fibrin deposition and fibrinoid arterial necrosis (**Figure 5C**). Serum anti-FcRn mAb levels indicated the presence of drug, although, as previously, circulating levels fell rapidly after 24h, and therefore were not captured following the initial administration of anti-FcRn 5 days before kidney transplantation (**Figure 5D**). Total circulating IgG levels reduced in response to treatment prior to kidney transplantation. However, despite a brief rise in circulating IgG on the first post-operative day, following repeated administration of anti-FcRn on POD 0 and POD 5, there was a reduction compared to pre-transplant baseline (**Figure 5E**). With respect to circulating DSA, there was an initial reduction from baseline levels, but after POD 4, and the increase from baseline was exponential (**Figure 5F**). IgM DSA showed a similar trajectory, albeit with a more limited reduction prior to transplantation (**Figure 5G**).

**Table 1:** Banff Scoring of rejected kidney allografts.

	t	v	i	ti	i-IFTA	g	ci	ct	cg	mm	cv	ah	ptc	Tubular Injury	Interstitial Plasma cells	Interstitial Neutrophils	Interstitial Eosinophils	Edema	Inclusions	PTC fibrin	Glomerular fibrin	Arteriole fibrin	Interstitial Hemorrhage	C4d (N.A. = not available)	**Diagnosis [ACR = acute cellular rejection, AMR = antibody-mediated rejection, TMA = thrombotic microangiopathy]**
K541	2	3	2	2	2	3	1	0	1b	3	0	0	3	1	0	0	0	1	0	0	1	1	1	1	**ACR, type 3**, with fibrinoid arterial necrosis & endarteritis. Findings also suspicious for **CAMR** (transplant glomerulopathy, peritubular capillaritis, glomerulitis, TMA-type findings with glomerular fibrin, fibrinoid arterial necrosis)
K722	1	0	2	2	2	3	1	1	1b	1	0	0	3	1	0	0	0	1	0	0	0	0	1	1	“Borderline changes” “suspicious” for ACR. Findings also suspicious for AMR (glomerulitis, peritubular capillaritis, & possible early transplant glomerulopathy)

Given the repeated administration of Rh anti-FcRn, both following the earlier 30mg/kg single infusion, as well as the subsequent repeat dosing of 60mg/kg IV, ADA were measured by ELISA. One of the animals demonstrated evidence of ADA in greater concentration from POD 1 (**Supplemental Figure 3** **)**, although the functional consequence of this antibody is unclear given the similar pattern of short survival and allograft AMR. To investigate the relative lack of efficacy of Rh anti-FcRn we first investigated whether the presence of Rh anti-FcRn mAb could be interfering with the concurrent RhATG therapy. We compared serum RhATG levels measured on d4/5 post-administration of RhATG in anti-FcRn treated animals (n=2) to animals who received Rhesus (n=7) or rabbit ATG (n=4) as part of a different experiment (In submission). There was no apparent difference in circulating RhATG concentration on POD4/5 (**Supplemental Figure 4**). Taken together, perioperative administration of anti-FcRn mAb successfully interfered with total circulating IgG maintenance *via* FcRn-mediated IgG recycling. However, it did not result in the reduction of circulating DSA (**Figure 5F**) compared to controls without anti-FcRn mAb treatment (**Supplemental Figure 5A**). This points to anti-RhFcRn being insufficient to reduce alloantibody produced by newly generated plasma cells as a consequence of rapid differentiation from the memory B cell compartment.

## Discussion

In this study, we demonstrate the first application of anti-FcRn in a sensitized rhesus macaque model to evaluate circulating DSA. Targeting the FcRn-mediated recycling mechanism in transplantation is extremely attractive, since an effective treatment has the potential to reduce the requirement for both plasmapheresis and high-dose IvIg (18, 32). As expected, anti-FcRn mAb treatment (30mg/kg) promoted a significant reduction of total IgG level as well as donor-specific IgG **(** **Figure 2** **)**. Circulating anti-FcRn mAb was only detectable on the PID 1, indicating rapid uptake and efficacy, which is in keeping with previous studies (15, 16). Interestingly, although the reduction of IgG DSA was less than total IgG at nadir (POD4: BFXM -47.8% ± 19.8, TFXM -59.4 ± 12.5, *vs.* 68% ± 10.6), it took longer for IgG DSA to return to baseline level. Given the natural decay of DSA after skin transplantation, it is uncertain whether the observed phenomenon truly represents an increased sensitivity of IgG DSA with respect to inhibition of the FcRn-mediated recycling or not, although compared to time-matched controls, the reduction of DSA at PID 28 was significant (**Supplemental Figure 1**).

As our results demonstrate, post-transplant there was incomplete reduction of the circulating the DSA IgG response. In part, this may relate to the construct of the Rh anti-FcRn mAb itself. The IgG1 isotype might be less effective at targeting the FcRn mechanism compared to rozanoliziumab, which is constructed with an IgG4 isotype. However, it should be considered that the direct relationship between NHP IgG isotypes with respect to their human IgG subtype corollary as well as their pathogenicity is unclear. However, it should be noted that an increased dose (60mg/kg, compared to the single dose of 30mg/kg) did not lead to a greater reduction of either IgG or DSA IgG from baseline, suggesting saturation. In the meantime, anti-FcRn mAb treatment showed the absence of any significant effect of circulating total IgM or DSA IgM **(** **Figure 3** **)**. IgM DSA is rarely measured in the clinical setting and is largely present at lower circulating levels, although since it is a more potent activator of complement, high levels of IgM DSA have been linked to aggressive AMR resistant to complement therapy in both humans and NHPs ( (33) Schmitz et al, under revision). As evidenced by this data, FcRn is an IgG-targeting mechanism, and therefore offers no protection in that setting.

Furthermore, given the non-specific nature of targeting the FcRn, as demonstrated with the observed decrease in CMV and anti-tetanus toxoid antibody responses **(** **Figure 4** **)**, the off-target effect with respect to protective immunity likely precludes prolonged usage. Even for patients with an otherwise intact immune system, such as those highly sensitized patients consideration should be given to the negative consequences of decreasing circulating protective antibody for any prolonged period of time. Alternatively, combining anti-FcRn treatment with administration of high dose IvIg may have the dual benefit of permitting the exogenous passive transfer of protective antibody, while additionally providing homeostatic pressure to reduce the rapid recapitulation of the recipients own alloantibody response.

Our results indicate that, in fact, the efficacy of the treatment, with respect to reduction of circulating total IgG, was maintained with repeated dosing post-transplant. However the paradoxical increase in DSA IgG post-transplant, whilst receiving repeated anti-FcRn treatment, potentially indicates that, in the sensitized recipient, the response to allograft itself drives a rapid DSA production likely from memory B cells (anamnestic response), which can easily overwhelm the effects of the anti-FcRn. Although it has been modelled that in states of greater IgG production, such as autoimmune disease, the efficacy of anti-FcRn therapy is likely increased (34) our speculation is plausible, since targeting FcRn-mediated IgG recycling results in the accelerated removal of IgG, but does not interfere with the production of IgG. In other words, blockade of FcRn is likely rendered ineffective in the setting of rapid or ongoing antibody production, such as is the case in active AMR.

Interference with other antibody-based therapeutics should be considered as another downside of using an anti-FcRn approach in transplantation. The widespread use of Thymoglobulin (ATG) and other monoclonal antibody–based treatments, such as rituximab or alemtuzumab, in addition to belatacept-based maintenance regimes, raise the concern that targeting the FcRn receptor would reduce the availability of circulating therapeutic antibody-based treatment, although it should be noted that our transplant data lack conclusivity with respect to any reduction of depletional effect of RhATG induction (**Supplemental Figure 5**), although early cellular rejection hints at an incomplete lymphocyte depletion at induction (**Figure 5**). Comparison of circulating RhATG levels in anti-FcRn treated animals and animals part of an another project receiving RhATG indicate no significant lowering of circulating RhATG levels on POD 4/5 (manuscript submitted, **Supplemental Figure 4**).

Despite this, targeting FcRn-mediated IgG recycling remains an attractive alternative to plasmapheresis with which to reduce pre-formed DSA, particularly when considering the many side effects from plasmapheresis—particularly cardiovascular and coagulation related—that serve to limit its utility for all transplant candidates. The question of how and how such a drug might be used in transplantation is therefore clearly of interest to the field. The most obvious possible use for anti-FcRn targeting is, therefore, potentially for sensitized patients awaiting a kidney allograft; however, the potential for a vigorous anamnestic response should be considered with respect to organ acceptance and immunosuppression options, while as described earlier, combination therapy with IvIg administration may serve to suppress the repopulation of the immunoglobulin compartment with DSA. Anti-FcRn treatment may also be of utility when combined with agents targeting Ab-producing cells, such as proteasome inhibitors (i.e. bortezomib, carfilzomib, etc.). In this context, giving anti-FcRn could rapidly reduce DSA in circulation, while proteasome inhibition would target and limit the source of additional and rapid DSA production which might otherwise overwhelm the efficacy of anti-FcRn.

To our knowledge, this is the first attempt to test the efficacy of anti-FcRn mAb on DSA in the transplantation setting. Our data clearly demonstrated potential of anti-FcRn mAb in reducing DSA, while also demonstrating its limitations, including a lack of ability to control DSA when accompanied by new production of IgG. Further investigation in how to use it in the clinical setting is required for translation of this agent in transplantation.

## Data Availability Statement

The original contributions presented in the study are included in the article/**Supplementary Material**. Further inquiries can be directed to the corresponding authors.

## Ethics Statement

The animal study was reviewed and approved by Duke University Institutional Animal Care and Use Committee (Duke IACUC# A153-18-06).

## Author Contributions

MM participated in research design, conduct of experiments, acquisition of data, analysis of data, and writing of the manuscript. WF, JY, and YB conducted experiments and acquired data. RS, ZF, and BS participated in surgical procedures, AK participated in research design and analysis of data. MH and SP participated in measuring anti-TT and anti-gB antibody level, ABF participated in conducting experiments and acquiring data (histology and histological grading). DM provided research product (anti-FcRn mAb), participated in research design and conduct of experiments. JK and SK participated in research design, conduct of experiments, analysis of data and writing of the manuscript. All authors contributed to the article and approved the submitted version.

## Funding

This work was supported by the National Institute of Allergy and Infectious Diseases of the National Institutes of Health as part of the Nonhuman Primate Transplantation Tolerance Cooperative Study Group under U19AI131471 (awarded to SK).

## Disclaimer

The content is solely the responsibility of the authors and does not necessarily represent the official views of the National Institutes of Health.

## Conflict of Interest

The authors declare that the research was conducted in the absence of any commercial or financial relationships that could be construed as a potential conflict of interest.

## Abbreviations

ADA, Anti-drug Antibody; APC, Antigen Presenting Cell; AMR, Antibody Mediated Rejection; BCR, B-cell Receptor; BFXM, B-cell Flow Crossmatch; CFCA, Calibration-free Concentration Analysis; DFPP, Double Filtration Plasmapheresis; DSA, Donor Specific Antibody; ESRD, End Stage Renal Disease; FcRn, Neonatal Fc Receptor; FXM, Flow Crossmatch; HLA, Human Leucocyte Antigen; IdeS, Imlifidase; PEX, Plasmapheresis; POD, Post-operation Day; MHC, Major Histocompatibility Antigen; NHP, Non-Human Primate; PBMC, Peripheral Blood Mononuclear Cell; PBS-T, Phosphate-buffered saline with Tween; TFXM, T-cell Flow Crossmatch; TMA, Thrombotic microangiopathy.
