# Supplementary material for: Measuring the Impact of Targeting FcRn-Mediated IgG Recycling on Donor-Specific Alloantibodies in a Sensitized NHP Model
Source: Front Immunol. 2021 Jun 2;12:660900. doi: 10.3389/fimmu.2021.660900 (PMC8207189; doi:10.3389/fimmu.2021.660900)
Supplement: Supplementary file 1 [file DataSheet_1.docx]

Supplementary Information for

**Measuring the Impact of Targeting FcRn-mediated IgG Recycling on Donor-Specific Alloantibodies in a Sensitized NHP Model**

**Authors:**

Miriam Manook^1^, Walter J. Flores^2^, Robin Schmitz^1^, Zachary Fitch^1^, Janghoon Yoon^1^, Yeeun Bae ^1^, Brian Shaw^1^, Allan Kirk^1^, Melissa Harnois^3^, Sallie Permar^3^, Alton B. Farris^4^, Diogo M. Magnani^2^, Jean Kwun^1,^†, and Stuart Knechtle^1,^†

^1^ Duke Transplant Center, Department of Surgery, Duke University Medical Center, Durham, NC 27710

^2^ Massbiologics of the University of Massachusetts Medical School, MA 02125

^3^ Human Vaccine Institute, Duke University Medical Center, Durham, NC 27710

^4^ Department of Pathology, Emory School of Medicine, Atlanta, GA 30322

# Corresponding author:

Dr. Jean Kwun *and* Dr. Stuart J. Knechtle,

Department of Surgery, Duke Transplant Center

Duke University Medical Center

207 Research Drive

362 Jones Building

Durham, NC 27710

E-mail: jean.kwun@duke.edu or stuart.knechtle@duke.edu

**Table of contents:**

**Supplemental Figure 1.** IgG DSA kinetics compared to Control (untreated) animals

**Supplemental Figure 2.** Peak (1^st^ & 2^nd^) DSA compared to Control (untreated) animals

**Supplemental Figure 3.** Differential ADA response peri-transplant

**
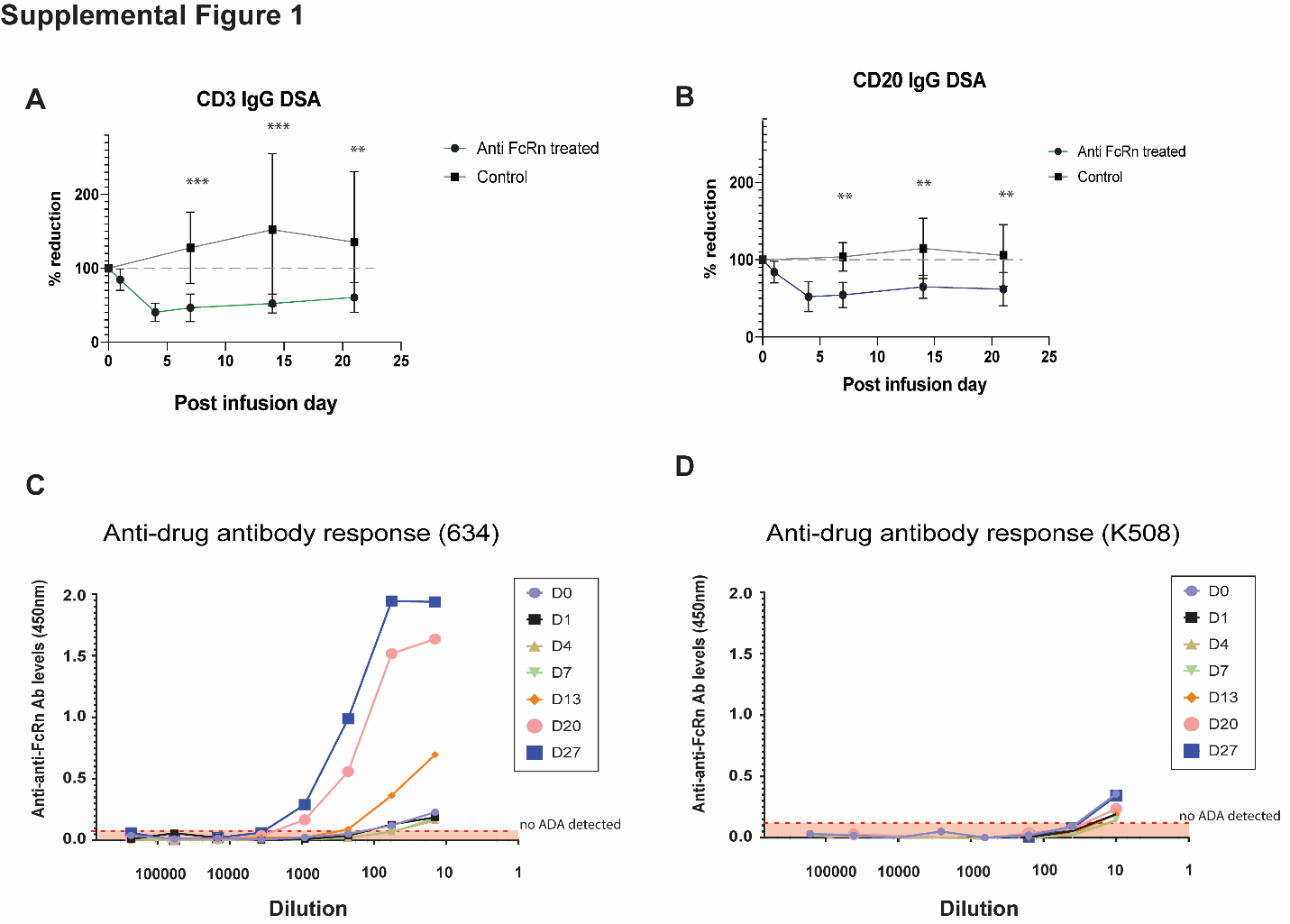
**

**Supplemental Figure 1.** Demonstrating percent reduction of (**A)** TFXM (CD3) and (**B)** BFXM (CD20) following anti-FcRn dosing (30mg/kg IV) comparing treated animals to time matched historic controls receiving no treatment. * indicates p<0.05; ** indicates p<0.001; *** indicates p<0.005.

Representative detection of anti-anti-FcRn (anti-drug antibody, ADA) levels by ELISA for (**C)** a positively responding animal (634) and (**D)** an unresponsive animal (K508).

**Supplemental Figure 2. (A)** Representative DSA MFI (CD3 or CD20) of animals from untreated historic control group and anti-FcRn treated pre-sensitisation, first peak (2 weeks following first skin transplant) and second peak (2 weeks following second skin transplant). Anti-FcRn treatment occurred following first peak, and prior to second skin transplantation. (B) Fold change of DSA (B cell flow crossmatch) for untreated and treated animals at first and second peaks, demonstrating no significant difference thus indicating an intact ability to generate DSA following antigenic stimulus. NS indicates no statistical significance.

_
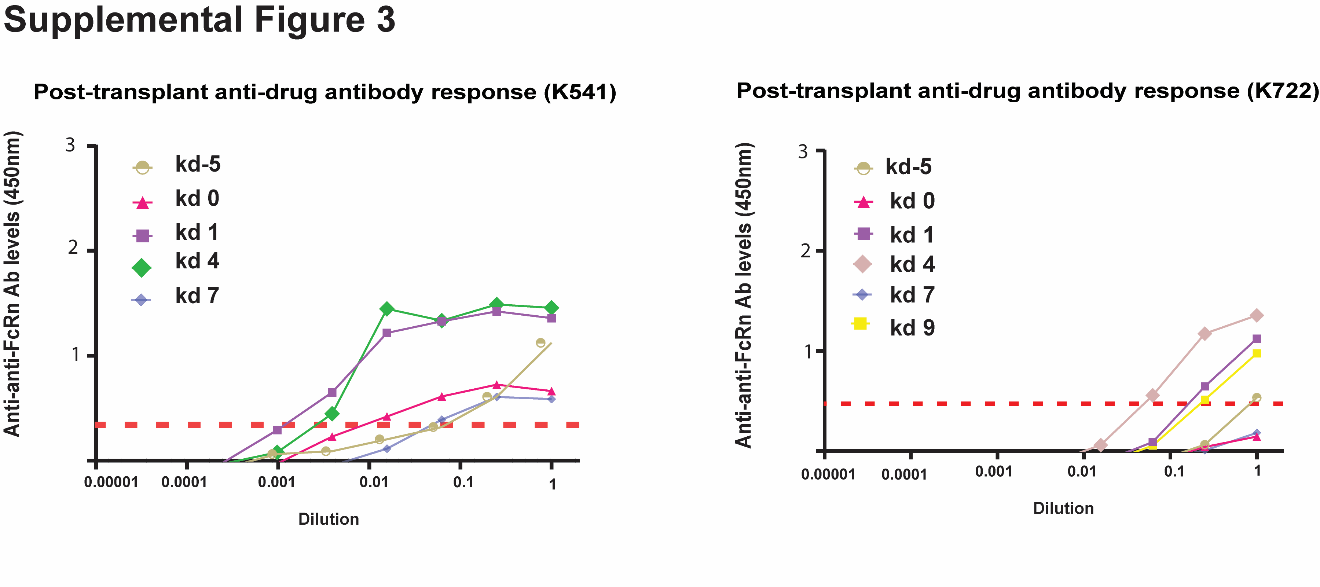
­­_

**Supplemental Figure 3.** Post-transplant anti-drug antibody (ADA) responses by ELISA in anti-FcRn transplanted animals, demonstrating evidence of ADA detectable prior to transplant (kd-5), with variable responses by animal.

**
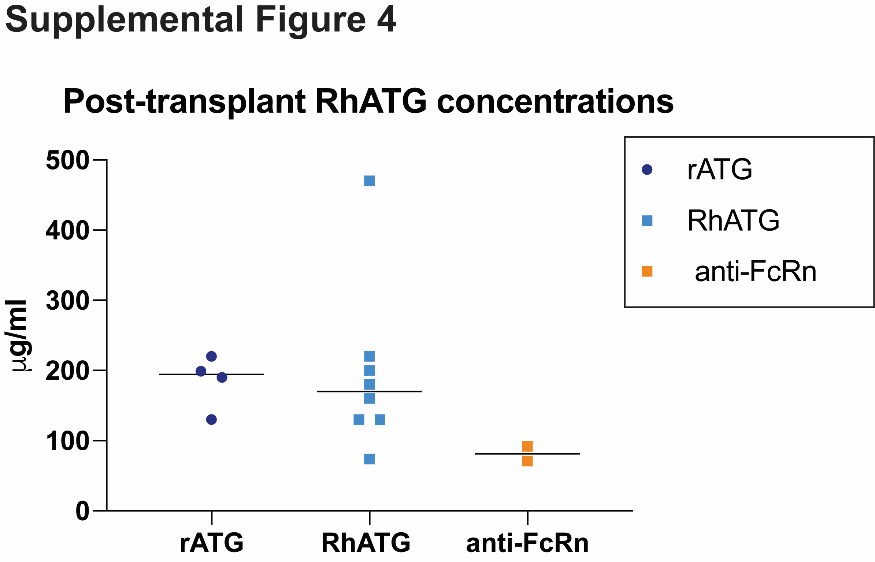
**

**Supplemental Figure 4.** Rhesus ATG levels detected in plasma on post-transplant d4 or 5, comparing animals treated with Rhesus ATG (RhATG, n=7), to those treated with rabbit ATG (rATG, n=4) with animals treated with RhATG and Rh anti-FcRn (anti-FcRn, n=2).


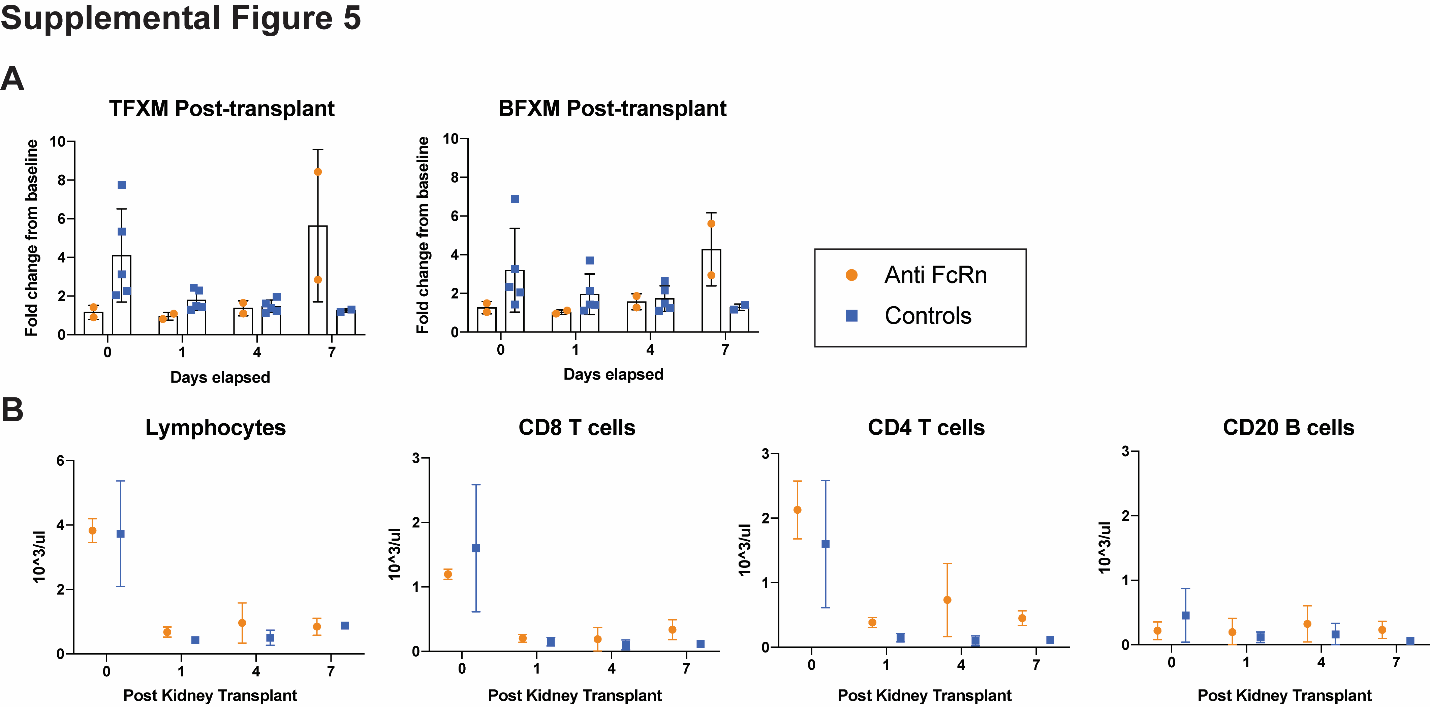


**Supplemental Figure 5.** Post-transplant comparison of kidney recipients between anti-FcRn treated vs. controls. **A.** Post-transplant donor specific antibody as measured by T- and B cell flow crossmatch (TFXM and BFXM). **B.** Absolute numbers of lymphocytes, CD4 T cell, CD8 T cell and CD20 B cells with (anti-FcRn mAbs; n=2) or without (controls; n=5) after transplantation. All animals received rhATG (4mg/kg daily) on post op day 0-4.
